# Political violence in democracies: An Introduction

**DOI:** 10.1177/00223433251351251

**Published:** 2025-08-22

**Authors:** Andrea Ruggeri, Ursula Daxecker, Neeraj Prasad

**Affiliations:** Department of Social and Political Sciences, Università degli studi di Milano, Italy; Department of Political Science, University of Amsterdam, the Netherlands; Department of Political Science, University of Amsterdam, the Netherlands

**Keywords:** Conflict, democracy, political violence

## Abstract

It is well established that democracies experience less political violence than autocracies. Paradoxically, however, this widely accepted fact has led scholars to overlook the existence of various forms of political violence *within* democracies. This special issue introduction article sees political violence as collective violence aimed at achieving political goals, encompassing electoral, ethnic, criminal, and terrorist violence. It reviews what we know about variation in political violence *across* democracies, which turns out to be surprisingly little. The article argues that normative preconceptions, rationalist theoretical traditions, and measurement challenges may explain gaps in our knowledge, such as insufficient attention to the strategies used by violent actors, the partisan and demographic determinants of support for violence, and the purpose of violence. We proceed to introducing the 14 special issue articles, which study political violence with cutting-edge methodologies in the three most democratic regions in the world. The individual articles advance research in four key areas: (1) strategies of violent actors to avoid the accountability constraints of democracy; (2) the actors sponsoring violence; (3) the effects of political violence in democracy; and (4) the debate on popular support for political violence. Addressing theoretical and methodological shortcomings in prior work, this introduction and special issue highlight that democracy – despite its many merits – was never quite as peaceful as it may have seemed.

*‘Beneath all the forms of polite society lies a stratum of potential violence which constitutes the ultimate test of the viability of social groups and institutions.’ (Nieburg, 1969: 16)*

## Introduction

In January 2021, a mob of over 2,000 people stormed the United States Capitol, violently disrupting the peaceful transfer of power in one of the world’s oldest democracies. The attack surprised many; until then, most research on political violence had focused on hybrid and authoritarian regimes in the Global South. Insofar as violence in the Global North was examined, it largely centered on nonstate actors challenging the status quo, such as terrorists. More recently, scholars have begun to explore political violence in advanced democracies (Costalli et al., 2024; Dancygier, 2023; Eady et al., 2023; Kalmoe and Mason, 2022; Kleinfeld, 2021; Krause and Matsunaga, 2023; Piazza, 2023; Riaz et al., 2024; Westwood et al., 2022). This special issue contends that key gaps remain. When and why do elites in stable democracies condone, incite, or sponsor political violence? How do they evade democratic accountability? And under what conditions do voters support or reject such violence? Drawing on diverse literatures and the contributions in this issue, we argue that democracy – despite its many merits – was never quite as peaceful as it may have seemed.

Our point of departure is democracy’s^1^ well-established ability to process conflicts peacefully (Przeworski, 2011, 2019; Schattschneider, 1960). Democratic institutions are generally seen as effective in constraining incumbents from the most severe forms of political violence, including interstate war among democracies, large-scale repression, and civil war (Davenport, 2007; Gleditsch, 1992; Hegre et al., 2001). This emphasis on democracy and peace, however, has contributed to political violence within democracies being relatively understudied – despite its continued existence. Violence against immigrants and other minority groups occurs routinely in democracies in the Global North and South. Concerns over violence against the democratic process itself, such as intimidation of voters, candidates, politicians, and election officials have also intensified in recent years. Moreover, some forms of political violence, such as terrorism or criminal violence, seem to disproportionately affect democratic regimes. While work has explored criminal, electoral, ethnic, and terrorist violence (for reviews, see Barnes, 2017; Birch et al., 2020; Chenoweth, 2013; Green and Seher, 2003), it has often done so in isolation, and without engaging its puzzling persistence in democracies. Hence, the core question we engage in this special issue is why and how political violence remains prevalent in democracies, which requires understanding how the sponsors of violence avoid accountability constraints coming from democratic institutions and voters. Paradoxically, the well-documented lower levels of political violence in democracies compared to autocracies may be why the field has neglected the continued existence of political violence *within* democracies. Hence, current approaches focus on explaining democracy’s pacifying effects rather than accounting for variation in political violence *across* different democratic settings.

The aim of our introduction is threefold. Our first aim is to conceptualize political violence and review what we know about its variation in democracies, which turns out to be surprisingly little. Second, we establish three reasons for why scholarship may have neglected to explore this variation; these are normative preconceptions, failing to theorize violence as a political process, and empirical approaches that favor the study of severe violence. Our third aim is to introduce the 14 contributions. The articles in this special issue contribute novel insights in four domains: (1) the strategies violent actors use to avoid the accountability constraints of democracy, (2) the actors engaging in violence, (3) the coercive and persuasive effects of violence, and (4) the debate about popular support for political violence.

## Defining political violence

Politics and violence are closely linked. Politics is the study of power, aiming to understand ‘who gets what, when, and how’ (Lasswell, 1936). Violence is one of the most common ways through which actors can exercise power and enforce order, aside from material or moral inducements (Bernhard and O’Neill, 2020; Etzioni, 1968). With states as the primary form of political organization, they have become the primary sponsors of violence aiming to enforce order (Davenport, 2007; Mars, 1975; Rummel, 1983; Schwarzmantel, 2010). At the same time, states’ monopoly over violence is often incomplete (Kleinfeld and Barham, 2018; Staniland, 2021), and violence can also be a means for nonstate actors to try and change existing power relations (Mars, 1975; Rapoport and Weinberg, 2001; Schwarzmantel, 2010).^2^ Most scholarship, then, conceives of political violence as the threat or use of physical force to enforce or disrupt a particular order.

Recognizing the importance of violence in politics, ample scholarship has studied it across a broad range of manifestations and practices. Yet this engagement has come at the expense of conceptual clarity or cohesion, with some scholarship studying larger conflicts with varying levels of violence, while others focus on particular instances of violence. For instance, there are extensive literatures on international war, civil war, and ethnic conflict. Typically, violence in these conflicts must cross a minimum threshold to qualify for inclusion; however, the conditions leading to these conflicts are often distinct from what explains patterns of violence within them (Brubaker and Laitin, 1998; Kalyvas, 2006).^3^ Informed by social movements literature and sociology, other scholars study political violence across a broad set of repertoires (De La Calle and Sánchez-Cuenca, 2024; Della Porta, 2013; Gutiérrez-Sanín and Wood, 2017; Tilly, 2003). This scholarship highlights that different forms of violence vary in targeting, frequency, and technique, and that this variation is worth studying (Gutiérrez-Sanín and Wood, 2017: 35). Finally, entire literatures studying particular instances have developed, including scholarship on repression, ethnic violence, electoral violence, terrorism, and criminal violence (Birch et al., 2020; Brubaker and Laitin, 1998; Chenoweth, 2013; Davenport, 2007; Green and Seher, 2003; Kalyvas, 2015). However, these literatures do not necessarily engage with each other, leaving unclear whether various forms have similar causes, how they relate to each other, or even what makes violence political in the first place (Kalyvas, 2019).^4^

We do not intend to resolve ongoing conceptual debates on political violence in this special issue. But since we are interested in understanding whether and how political violence changes in democracies, we require a definition that can identify a broad set of potential manifestations, including violence that may not always be identified as political, particularly in democracies. Debates around criminal violence, for example, are increasingly challenging narrow interpretations of such violence as purely ‘private’ and apolitical; acknowledging that these interpretations benefit state actors – including democratically elected ones – who collude with criminals (Durán-Martínez, 2017; Lessing, 2017). Moreover, it is important that we capture violence by state and nonstate actors; that is, violence that aims to preserve or disrupt the status quo. Following Costalli et al. (2025) in this special issue, we define political violence as ‘the use of collective violence aiming to achieve a political goal, which involves altering (or preserving) the institutional setting and the existing power relations among the relevant political actors of a community’ (Costalli et al., 2025: 1598).

## Political violence in democracies: What do we know and what is missing?

The number of countries with democratic institutions increased dramatically in the 20^th^ century. During the Cold War, conflict scholars did not pay much attention to the spread of democracy; this changed with the collapse of the Soviet Union. The most important finding from literature on the relationship between democracy and political violence is one of relative absence; that is, democracy seems to exert a large and robust negative effect on war, armed conflict, and repression. The oldest and strongest of these findings is the absence of interstate wars between democracies, a relationship attributed the law-like status rare in the social sciences (Babst, 1964; Gleditsch, 1992; Mitchell, 2002; Rummel, 1983). Subsequent research on civil war documented a pacifying effect of democracy, establishing the existence of a ‘democratic civil peace’ (Cederman et al., 2010; Gleditsch and Ruggeri, 2010; Hegre et al., 2001). Extending these findings to repression, work has found that state-sponsored coercion diminishes in democracies, although only once democracy reaches a high threshold (Davenport, 2007; Davenport and Armstrong, 2004). Theoretical explanations for these findings – dating back to the Enlightment, French and American revolutions, and works such as Kant’s *Perpetual Peace* (1795) and de Tocqueville’s *Democracy in America* (1835–1840) – have emphasized the institutional and normative foundations of the more peaceful orientation of democracies. Institutional accounts emphasize that democratic institutions raise the cost of using violence for state actors because they can be punished for aggressive behavior. Simultaneously, the institutions of democracy give nonstate actors nonviolent channels for participation and contestation, which help create normative commitments towards peaceful dispute resolution (Rapoport and Weinberg, 2001). This system works if political forces have institutionalized access to the representative system and incentives to engage within it – most crucially, through adherence to fixed-term electoral procedures (Przeworski, 2011). Furthermore, even in the face of unfavorable electoral outcomes, democratic actors must maintain incentives to tolerate the results (Przeworski, 2018). Democracies, therefore, are founded on the principle of managing incompatible preferences and interests peacefully.

Other work, however, raises questions about the presumed peacefulness of democracies.^5^ Largely forgotten, modern political science’s engagement with political violence dates back to the late 1960s and early 1970s in the United States, a period marked by heightened protest and strike activity but also characterized by frequent use of violence by state and nonstate actors (Graham and Gurr, 1968). Social and political conflict was not confined to the United States; in affluent European countries, including Italy and France, average strike volume peaked in the late 1960s (Sánchez-Cuenca, 2019: 17). Moreover, research on terrorism has shown that democracies are more often targeted with violence than stable authoritarian regimes (Chenoweth, 2013; Gaibulloev et al., 2017).^6^ It is notable, then, that European countries struggled with political violence in times when democratic politics was also at its most progressive. Work on criminal violence similarly suggests that it can flourish rather than diminish in democracies (Arias and Goldstein, 2010; Davies et al., 2024; Lafree and Tseloni, 2006; Nussio, 2024). In Latin America, numerous studies have shown that criminal violence spiked precisely when democracy became more consolidated (Arias and Goldstein, 2010; Cruz, 2011; Trejo and Ley, 2018). Even more puzzling is the fact that democracy and its practices can become the target of political violence. As work on electoral violence has shown, incumbents and opposition routinely resort to intimidation and violence to influence the electoral process (Birch et al., 2020; Dunning, 2011; Harish and Little, 2017; Höglund, 2009).

Why, when, and how does ethnic, criminal, electoral, or terrorist violence continue to exist in democracies? There is surprisingly little research to tell us why accountability groups, including democratic institutions and the public, fail to protect democracy from violent threats. Theoretical literature explains that peaceful equilibria break down when groups’ peak preferences diverge significantly and the perceived cost of deviation from these preferences increases (Coser, 1964; Przeworski, 2018). Such polarization is likely when democracy fails to adjudicate on prevailing divisive issues or remains impervious to new political actors, leading to a persistent misalignment between dimensions of contestation and social preferences. Empirically, this implies that increasing interparty polarization could induce elites to incite or sponsor political violence (Bartels, 2023; Bartels et al., 2023; Levitsky and Ziblatt, 2018). Empirical findings, however, are mixed. While some studies find that growing polarization is associated with political violence (Kalmoe and Mason, 2022; McCoy et al., 2018), others fail to establish a relationship (Berntzen et al., 2024). A related literature has explored variation in citizens’ willingness to punish elites for engaging in political violence. Empirical findings, though, are contradictory; with some work showing strong condemnation for political violence, including from co-partisans (Alcorta et al., 2020; Gutiérrez-Romero and LeBas, 2020; Rosenzweig, 2021, 2023; Westwood et al., 2022), while others find no relationship or increased support for violent co-partisans (Daxecker and Fjelde, 2022; Daxecker and Prasad, 2025a; Siddiqui, 2023). Finally, prior work has shown that intense electoral competition, majoritarian electoral institutions, and electoral incentives for rent-seeking increase the risk of electoral and criminal violence in the Global South (Daxecker and Rauschenbach, 2023; Fjelde and Höglund, 2016; Klaus, 2020; Trejo and Ley, 2018), but we do not know if these patterns extend to democracies more broadly.

## Seeing like a democratic state: Gaps in the study of political violence in democracies

We note that debates in emerging work on violence in democracies are often framed around measurement or the context-specificity findings. We argue instead that scholarly inquiry may suffer from more fundamental blind spots. In this section, we explore three reasons for these gaps in our knowledge.

### Normative preconceptions: Othering political violence

A first potential source of bias are researchers’ normative commitments. The global rise of democracy and its virtues – especially its presumed peacefulness – could have led researchers to treat political violence as an aberration, as something that is *sui generis* or an outlier that may not deserve independent study.^7^ This might be why recent studies on the US depict political violence neutrally and without information about perpetrators or targets (Armaly and Enders, 2024; Uscinski and Parent, 2014). Yet when violence is presented without political context, precisely the dynamics that may shape institutional or popular support are lost. Normative preconceptions can also explain why work has sometimes assumed that citizens disapprove of political violence, rather than making this an empirical question. However, it is well known that partisan and other group-level biases drive most political attitudes (Graham and Svolik, 2020; Kunda, 1990; Taber and Lodge, 2006), and it is not clear why support for violence should be different. Citizens typically lack access to politically neutral accounts of violence; biased or incomplete information together with pre-existing partisan, ethnic, or religious biases could lead to substantial gaps between disapproval of political violence in the abstract and citizens’ willingness to hold members of in-groups accountable.^8^ Yet research designs depicting violence in neutral terms may not capture the geographically, politically, and demographically concentrated determinants of popular support (Armaly and Enders, 2024; Uscinski and Parent, 2014). Recent work on India, Germany, and the US shows that designs considering factors such as perpetrator and target identity show more substantial support for political violence (Dancygier, 2023; Daxecker and Prasad, 2025a; Kalmoe and Mason, 2022; Westwood et al., 2022),^9^ hence, evaluating whether support for violence demands attention to who supports violence, where they are located, how densely concentrated they are, and how central this support is for political behavior.

### Rationalist biases: Objectifying political violence

A second reason for gaps in our knowledge could come from rationalist traditions coupled with the above normative dispositions.^10^ Rationalist theorizing implies that material factors exogenously determine the preferences and action tendencies of actors (Elster, 1998), and therefore function as primary causes of political violence. Yet as constructivist and critical scholarship reminds us, theories are constructed and always represent someone for some purpose (Brass, 1997; Cox, 1981). Powerholders – including democratic ones – can define some forms of violence as apolitical, such as criminal violence (Barnes, 2017; Kalyvas, 2015), or even deny that practices are violent, such as harsh policing or strict immigration policies (Dancygier, 2010; Dancygier and Laitin, 2014; Eck et al., 2021; Hirschfield, 2023).^11^ Sensitive and controversial events such as political violence are especially vulnerable to social and political interpretations (Fiske and Rai, 2014; Jackman, 2002; Schmidt and Schröder, 2001). Political violence is often surrounded by intense speculation about ‘what really happened’, and powerful actors such as politicians can dominate narrative interpretation and legitimize violence as a response to a threat, injustice, or provocation (Brass, 1997, 2005; Bulutgil and Prasad, 2023; Daxecker and Prasad, 2025a, 2025b; Klaus, 2020; LeBas, 2006; Nussio and Clayton, 2023; Weaver, 2019). Political entrepreneurs do not take support as a given, but instead treat individual violent events as laboratories and opportunities to inoculate support for violence and test the limits of popular support for violence. This expost political articulation of violent events helps constitute the support of co-partisan elites and voters.^12^ Rationalist scholarship may not always be sufficiently attuned to these power dynamics, and be entrapped in a ‘conservative bias’ (Stoppino, 1973).^13^ The ongoing war in Gaza and debates about what to call the violence and whether Israel is still a democracy are cases in point.

### Challenges in measurement: Counting violence

Gaps in scholarship could also stem from positivist epistemology common in contemporary political science, including peace research. Positivist approaches – especially when involving quantitative methods – predispose our research towards violence that is visible and can be measured, which could introduce various forms of systematic measurement error. First, data on political violence often come from governments or media. While most researchers would be suspicious of these data in authoritarian regimes, they may be more willing to rely on it from democracies. Contributions on political violence in Germany, for example, rely on government-sourced data, and have adopted legal rather than analytical categories to describe the violence (Riaz et al., 2024). Yet democratic governments may also have incentives to under- or misreport some violence (Dietrich and Eck, 2020; Gunther and Mughan, 2000). Similarly, while media in democracies will report more openly and critically of government than autocracies, they are not immune from government pressure (Baum and Potter, 2008). Second, an emphasis on what can be measured explains why more severe forms of political violence – which are more prevalent in non-democracies – have received more attention than non-lethal or hidden forms (Krause, 2018; Steele, 2018). Yet less easily observable forms of coercion may be just as common in democracies as in authoritarian regimes, and may be just as pernicious in normative implications. A recent study of India – relying on list experiments and fine-grained local news – finds that electoral intimidation remains widespread in some Indian states (Daxecker et al., 2024); this is surprising given India’s high rankings on measures of electoral competition (Ding and Slater, 2022). These biases may also be why literature on electoral violence, despite being interested in the relationship between democratic processes and political violence, has overlooked violence in industrialized democracies (Birch et al., 2020; Daxecker et al., 2019). Third, positivist research is predisposed towards political violence in the present. Past issues of this journal indicate that current events influence which forms of political violence are studied, moving from international conflict in the Cold War, to civil war and terrorism in the post-Cold War period. Presentist bias stems from publishing incentives and political context, leading scholars to focus disproportionately on contemporary political violence, thereby narrowing their temporal perspective and generating abstract conceptualizations based on recent events. While presentist bias would not only affect the study of political violence in democracies, electoral cycles and high-speed media cycles – crucial dynamics for a skewed focus on the present – are much more prevalent in democracies, predisposing them towards presentism (Hartog, 2015).

Normative preconceptions about violence as universally condemned, an underappreciation of interpretive processes surrounding the politicization of violence, and measurement challenges in studying morally sensitive topics point to noteworthy gaps in our understanding of political violence in democracies. These gaps may be why prior work has not paid enough attention to the strategies used to engender elite and popular support for violence, the motives of actors behind the violence, and the partisan, social, and contextual foundations of the violence. Our special issue articles offer novel analytical insights and significant empirical findings for an emerging research agenda on political violence and democracy. We now turn to introduce these contributions.

## Making violence in democracies visible: Contributions to the special issue

Our special issue reflects the global distribution of democracies. Twelve of 14 contributions cover countries in Europe, North America, and Latin America, which are the three regions with the largest number of liberal and electoral democracies in the world. Three articles study India and Nigeria, the two most populous democracies in Asia and Africa.^14^ The special issue represents state-of-the-art methodological approaches, including fieldwork, narrative analysis, surveys and survey experiments, difference-in-difference designs, and analyses of innovative data such as social media discourse and cell phone data. Substantively, the articles in the special issue address the blind spots identified above. We advance research in four areas: (1) strategies of violence, (2) the actors engaging in political violence, (3) the effects of violence, and (4) popular support for violence in democracies.

### Strategies of violence: Delegation, depoliticization, and legitimization

Actors sponsoring political violence in democracies must avoid international and domestic accountability groups that could punish them for violence. Our contributions highlight three strategies political elites use to avoid these costs. First, those sponsoring violence can delegate it to local units or nonstate actors; that is, they can increase the distance between them and those perpetrating the violence to plausibly deny responsibility. Existing literature has explored the delegation of violence to state-sponsored armed groups (Carey and Mitchell, 2017; Carey et al., 2016; Jentzsch et al., 2015); but as Fubara (2025) and Uribe (2025) demonstrate in this special issue, delegation is especially common when incumbents target the democratic process, i.e. elections as key moments when domestic and international actors pay most attention. Contributions also highlight that delegation goes beyond actors to lower-level political entities. Articles by Das (2025) and Fubara (2025) show that ostensibly democratic reforms such as decentralization can instead empower local incumbents to behave like ‘decentralized despots’ (Mamdani, 2018). In Nigeria, subnational incumbents recruit from existing armed groups or even create them (Fubara, 2025; see also Klaus and Turnbull, 2025). In India, local party networks are transformed into networks of coercion targeting voters and candidates (Das, 2025).

A second strategy is to depoliticize violence. This strategy aims to remove violence, both in terms of narrative and actions, from the political realm. Facts around violence are often highly contested (Brass, 1997; Kalyvas, 2003) and this strategic ambiguity can be exploited by powerful actors reinterpreting it as non-political, denying links between violent actors and politicians (Schedler, 2022). Several articles in our special issue confirm the depoliticization of criminal violence in Latin America (Albarracín et al., 2025; Córdova and Tiscornia, 2025; Masullo et al., 2025). Moreover, Albarracín et al. (2025) find that incumbents not only deny collusion with organized criminals, but also repress civil society activists resisting this collusion. Demonstrating how criminal violence reinforces democratic decline, Córdova and Tiscornia (2025) and Masullo et al. (2025) show that exposure to violence makes citizens supportive of heavy-handed government responses. Depoliticization and the delegation of violence can go hand in hand; in contexts such as Colombia, Uribe (2025) shows that the boundaries between criminal actors, the state, and armed groups are fluid.

Third, actors can legitimize violence as justified or even necessary to respond to a threat or injustice. Like depoliticization, this strategy is possible because violence often creates intense speculation about ‘what really happened’. Klaus and Turnbull (2025), Viganò et al. (2025) and Krakowski and Morales (2025) show that powerful actors such as party elites can portray violence as necessary to respond to alleged threats or injustices by an unpopular target, such as an out-group or institution with pre-existing grievances. For example, before and after 6 January 2021, Republican elites in the United States sponsored false claims about election fraud and the alleged involvement of the ‘deep state’ that helped legitimize violence. Extending these findings to rhetoric, Meye (2025) finds that narratives of election fraud by Republican elites in the United States mobilized citizens to support the party. Legitimization in the US case has nuanced effects; while violent rhetoric by Republican elites reduces engagement by co-partisans (Krakowski and Morales, 2025), this backlash does not extend to co-partisans’ voting behavior or offline mobilization (Meye, 2025). Focusing on Europe, Viganò et al. (2025) and Carreras et al. (2025) present evidence in favor of the effectiveness of legitimization; Viganò et al. (2025) show that extreme right-wing parties benefited from violence against a perceived threat in interwar Italy, while Carreras et al. (2025) establish that hostile and xenophobic anti-immigrant discourse can increase electoral support for right-wing parties in Europe today. In a contribution on the ongoing war in Ukraine, Bakke et al. (2025) show that exposure to violence reduces citizens’ commitment to minority rights but has no clear effect on other democratic principles – highlighting both the potential for violence to deepen in-group/out-group divides and the resilience of democratic norms.

### Actors sponsoring violence

State and nonstate actors engage in political violence in democracies (Costalli et al., 2025); state actors in democracy can resort to violence rather than accommodating challenges to political order. Compared to their nonstate counterparts, incumbents have greater resources to organize violence; as our contributions show, this is why incumbents can use delegation, depoliticization, and legitimization to try and defend existing political structures. The orders defended by incumbents vary; for example, Rivera et al. (2025) explore violence aiming to maintain the patriarchy, while Albarracín et al. (2025) study repression of civil society actors protesting crime-state collusion, and Das (2025), Fubara (2025), and Uribe (2025) examine incumbent violence to sustain rent-seeking opportunities.

In contrast, nonstate actors use violence to contest existing orders. Compared to incumbents, they have fewer strategies at their disposal. They cannot delegate or depoliticize violence because these strategies aim to make violence invisible, which requires substantial resources and access to a coercive apparatus. This helps explain why nonstate actors with anti-systemic goals, such as terrorist groups, use violence sparingly and against highly symbolic targets to speak to an audience (Costalli et al., 2025). The aim of violence may be to elicit an overreaction from the government, which could persuade some members of the audience to support the group (Kydd and Walter, 2006). For nonstate actors with intrasystemic goals, such as opposition parties, the primary strategy is to legitimize violence. By sponsoring violence against an unpopular target, such as immigrants, other minorities, or allegedly corrupt institutions, opposition parties hope to attract new supporters (Carreras et al., 2025). This explains why opposition politicians, especially after losing elections, might be prone to violent and hostile rhetoric against unpopular targets (Krakowski and Morales, 2025; Meye, 2025).

### The effects of violence: Coercion or persuasion?

Regarding its effects, conventional wisdom is that political violence is primarily a tool of coercion; that is, it aims to alter the behavior of those targeted with it, or those identifying with the victims. Several articles in the special issue confirm coercive effects. For example, the presence of violent actors reduces electoral contestation, either eliminating opposition candidates from competing (Das, 2025), or increasing the vote share of parties affiliated with violence specialists (Uribe, 2025). Coercion also has gendered dimensions; Rivera et al. (2025) demonstrate that killings of female social activists reduce women’s participation in politics. Moreover, coercion undermines people’s faith into key aspects of democracy, reducing support for minority rights (Bakke et al., 2025), while increasing support for militarized politics (Córdova and Tiscornia, 2025; Masullo et al., 2025).

Yet other articles challenge the emphasis on coercion. Klaus and Turnbull (2025) describe how processes of narrative construction accompany violence sponsored by politicians. These narratives aim to persuade supporters rather than coerce opponents. Similarly, Meye (2025) and Carreras et al. (2025) explore the use of hostile and violent rhetoric, finding that it can help mobilize co-partisans sympathetic to this rhetoric. These findings imply that democratic institutions may impose an upper bound on coercion in wealthy industrialized democracies, making violence with persuasive goals more attractive in comparison.

### The debate on popular support for violence

The articles in this special issue speak to unresolved debates on popular support for political violence mentioned earlier in this introduction. Several articles challenge the assumption of a public that universally condemns violence; for example Córdova and Tiscornia (2025) and Masullo et al. (2025) show that citizens support violent government policing once violence is stripped of its political meaning. Moreover, some articles find that when party violence is legitimized through narratives of threat and injustice, co-partisans support violent parties (Carreras et al., 2025; Klaus and Turnbull, 2025; Meye, 2025; Viganò et al., 2025). These findings confirm the importance of processes of politicization in our empirical studies.

Yet other articles demonstrate the resilience of some democratic principles when citizens are confronted with violence. Bakke et al. (2025) find that Ukrainians exposed to quite severe violence do not trade off their support for free and fair elections. Others show similarly nuanced findings; for example, citizens exposed to crime in Brazil do not reduce their support for democracy overall even though they support unlawful enforcement (Masullo et al., 2025), and respondents in Mexico update support for militarization when collusion between criminals and state actors is made salient (Córdova and Tiscornia, 2025). These contributions highlight that even under duress, democratic citizens sometimes possess the capacity to cope with and address political violence through democracy’s unique mechanisms of conflict management and procedures.

## Concluding remarks

We conclude by revisiting Nieburg’s (1969) sharp observation that the veneer of a polite society – and he had in mind democracies – conceals a bedrock of potential violence. Our introduction has explored the intricate and often paradoxical relationship between democracy and political violence. We conclude with implications and suggestions for future research. First, to transcend ingrained biases, research must challenge conventional avenues of academic inquiry. Methodological rigor must be coupled with a critical self-awareness to counter theoretical and normative predispositions. Second, the practical realities of democratic governance require continuous attention to the tenuous equilibrium between (non-violent) conflict and democracy. Democracy, by its very nature, fosters contestation, yet excessive violent conflict can undermine its foundational principles. The inherent tensions between electoral competition and the safeguarding of minority rights exemplify this delicate balancing act. Third, while our special issue has focused on incentives for residual violence in democracies, more research on exogenous factors undermining it is needed. There is growing evidence of the tensions between democracy and late-stage capitalism (Boix, 2019). The Latin American experience with democracy – characterized by the precarious, violent bargains between political elites and criminal organizations illustrated in our special issue – serves as a stark reminder of these structural and non-straightforward challenges. Finally, the international dimension of democracy – and its behavior – profoundly shapes the tolerance of violent discourse and actions. The growing normalization of violent conflict within longstanding democracies such as India or the United States, alongside their involvement in foreign interventions, raises concerns about the stability of the democracy–conflict dynamic. This special issue underlines the requirement of examining the changing dynamic between democracy and violence in a globalized world. The future of democratic societies depends on this understanding. Rather than a flaw of democracy, we should see this continued struggle as a defining feature.

## Notes

1. We cannot tackle the debate on defining democracy in full here, but we can briefly summarize it as a debate between minimalist/maximalist definitions and along procedural /substantive features of democracy. Przeworski (1999) defended and defined a democracy as ‘a system where rulers are selected by competitive elections’, yet we believe that to have free and fair elections several elements present in a maximalist definition – safeguard for minorities – are necessary. V-Dem (Lindberg et al., 2014), instead of providing a single definition, describes five ideal-types: electoral, liberal, participatory, deliberative, and egalitarian. Classics are discussed by Sartori (1987), a more recent discussion by Munck (2016) and a discussion of democracy as moving target by Markoff (2011).

2. As Davenport (2007) notes, scholarship has prioritized political violence by nonstate actors rather than the state itself, despite the fact that state violence is more common and severe.

3. Moreover, violence within these conflicts can fluctuate widely across space and time (Kalyvas, 2006).

4. Kalyvas (2019) organizes various forms of political violence based on its perpetrators and targets, distinguishing each based on whether they are state or nonstate entities.

5. The process of democratization can also increase the risk of various forms of political violence (Mansfield and Snyder, 2007) and past political violence can also affect patterns of democratic practices (Costalli and Ruggeri, 2015, 2019), but both issues are beyond the scope of our special issue.

6. According to data on domestic terrorism victims, from 1965 to 2005, Western Europe recorded more than 4,500 victims in over 3,000 lethal attacks (De la Calle and Sánchez-Cuenca, 2011). Trends for more recent periods, however, challenge the greater vulnerability of democracies, showing instead that ‘autocracies and transitioning states have had higher numbers of attacks with more fatalities than democracies’ (Chenoweth, 2025).

7. Researchers studying political violence are often based in democratic countries in the Global North, which could strengthen these normative biases. For systematic explorations, see Phillips and Greene (2022).

8. For example, Daxecker and Prasad (2025a) show that in India, two-thirds of respondents say that political parties engage in violence, but only one in five attribute it to their own party.

9. In India, Daxecker and Prasad (2025a) find that 37% of respondents support political violence when targeted against out-groups. While lower on average, there is substantial support for political violence even in very wealthy democracies. In the US, surveys show that over 10% of respondents justify violence when the perpetrator is a co-partisan (Kalmoe and Mason, 2022; Westwood et al., 2022). In Germany, between 14% and 19% of the population supports hate crimes (Dancygier, 2023).

10. Rationalist ontology remains prominent in the study of political violence, including in this journal.

11. Some work goes a step further, and defines structural inequalities and cultural practices as structural and cultural violence, respectively (Galtung, 1969, 1990). To avoid conceptual stretching, our special issue is focused on political violence as observable instances of behavior producing injury and harm, while still recognizing that violence is socially and politically malleable.

12. Research designs that omit processes of legitimization therefore remove a critical aspect for measuring support for political violence. This implies testing attitudes towards violence after the processes of political legitimization and sorting are complete.

13. Among audiences favorable to nonstate actors challenging the political order, a similar bias in favor of rebels may also exist (Stoppino, 1973).

14. In total, the issue contains 14 articles. Klaus and Turnbull (2025) covers the United States and Nigeria.
